# Metformin-Based Combination Approaches for Triple-Negative Breast Cancer

**DOI:** 10.3390/pharmaceutics17050558

**Published:** 2025-04-24

**Authors:** Zaid Sirhan, Aya Abu Nada, Nadeen Anabtawi, Anita Thyagarajan, Ravi P. Sahu

**Affiliations:** 1 Department of Radiation Oncology, The Ohio State University Comprehensive Cancer Center, Columbus, OH 43210, USA; zaid.sirhan@osumc.edu; 2 Department of Pharmacy, Sidra Medicine, Doha P.O. Box 26999, Qatar; aabunada@sidra.com; 3 Division of Pharmaceutics and Pharmacology, College of Pharmacy, The Ohio State University, Columbus, OH 43210, USA; anabtawi.7@osu.edu; 4 Department of Pharmacology and Toxicology, Boonshoft School of Medicine Wright State University, Dayton, OH 45435, USA; anita.thyagarajan@wright.edu

**Keywords:** metformin, triple-negative breast cancer (TNBC), drug repurposing, cell signaling pathways, cancer therapy

## Abstract

Numerous anti-diabetic medications, including metformin, have been explored for their anticancer effects because of the substantial correlation between diabetes and cancer incidence. Metformin has recently gained interest for its anticancer effects against malignancies such as breast cancer, one of the leading causes of death among women worldwide. The cancer-related characteristics of cell proliferation, invasion, migration, and apoptosis are all targeted by metformin. Among breast cancer patients, triple-negative breast cancer (TNBC) is linked to an increased risk of early recurrence and metastases and has poor prognosis. In addition, TNBC has fewer treatment options compared to other breast cancer subtypes because it lacks hormone receptors and human epidermal growth factor receptor 2 (HER2), and it often develops resistance to available treatment options. The current review highlights the recent updates on the mechanistic insights and the efficacy of metformin and metformin-based approaches for the treatment of TNBC. We logically discuss the experimental evidence from the in vitro and in vivo studies exploring metformin’s effects on metabolic pathways, and then its combination with other therapeutic agents, targeting cell signaling pathways, and approaches to enhance metformin’s effects. We also present clinical studies that underscore the beneficial outcomes of metformin or its combination with other agents in TNBC patients.

## 1. Introduction

Breast cancer (BC) is one of the most commonly diagnosed malignancies in women in the world, and the second leading cause of cancer deaths among women [1,2,3,4]. BC is categorized into different molecular subtypes based on the prognosis and gene expression profiles [5,6,7]. One of those is triple-negative breast cancer (TNBC), which is defined by the lack of estrogen (ER) and progesterone (PR) hormone receptors, and human epidermal growth factor receptor 2 (HER2) [8,9]. TNBC accounts for approximately 10–15% of all BCs and exhibits poor prognosis and strong invasiveness, and often develops resistance to available therapeutic options [10,11,12]. Importantly, BC patients often present with comorbid conditions such as type 2 diabetes (T2D).

Notably, over the past 20 years, the prevalence rates of T2D have substantially grown, and the incidence is rising worldwide. Between 1990 and 2017, the prevalence of T2D increased from 11.3 million to 22.9 million, and by 2025, 26.6 million cases are anticipated [13]. Compared to nondiabetic individuals, T2D patients have an increased risk of developing liver, pancreatic, endometrial, colon, and BC, with the latter’s risk being exceptionally raised by T2D in 10% to 20% of the cases [14,15]. Given the importance of interconnected metabolic pathways in the development of human malignancies, repurposing anti-diabetic medications is gaining pace in cancer research, including for the treatment of BC [16,17,18,19,20].

Of significance, scientific research backed by the accumulating epidemiologic evidence encouraged investigation into the potential shared biological pathways between T2D and BC and the development of therapeutic possibilities for treating BC [21,22,23,24]. The common genetic pathways that are shared between T2D and BC are insulin-like growth factor 1 (IGF-1), adipokines and the release of pro-inflammatory adiponectin/leptin, and a weakened immune response brought on by increased levels of pro-inflammatory cytokines like tumor necrosis factor-α (TNF-α) [25,26,27]. To emphasize, insulin is a peptide hormone secreted by pancreatic beta cells in response to hyperglycemia. When insulin binds to its receptor, it promotes the phosphorylation of insulin receptor substrate (IRS1) and subsequently activates the phosphatidylinositol 3-kinase (PI3K)/protein kinase B (AKT)/mammalian target of rapamycin (mTOR) signaling cascade [17,28,29,30]. In addition, insulin and hyperglycemia stimulate IGF1 production, and the binding of IGF1 to its receptor activates the downstream signaling pathways that involve PI3K/AKT/mTOR and the RAS/RAF/mitogen-activated protein kinase (MAPK) pathways [30,31,32]. These pathways are frequently dysregulated in tumor cells and provide a mechanistic link between insulin/IGF1 and the onset and progression of cancer. Among other factors, tissue inflammation, increased cancer cell motility and invasion, reactive oxygen species (ROS) production, and angiogenesis have been shown to be linked with BC progression [17,28,29,30,33].

Importantly, insulin receptor (IR) and IGF1-R expression levels in BC cells are markedly higher than in healthy breast tissues. BC patients with high insulin levels have bad prognosis, and chronic hyperinsulinemia results in lowering IGF Binding Protein 1 (IGFBP-1) levels and increasing the bioactive concentrations of IGF1 [34]. Other important cell signaling molecules shared between T2D and BC are adipokines or adipocytokines. Recent research has focused on defining the roles of adipokines, and hormones secreted by adipose tissue, in oncogenesis. As obesity is associated with an increased risk and higher chance of developing BC [35], adipokines in obese patients can directly affect metabolic pathways like the Janus kinase–signal transducer and activator of transcription (JAK-STAT) or PI3K or alter the tumor microenvironment (TME) [36,37]. Furthermore, a rising number of adipokines, over 10 in total, have been linked to BC [38]. Adiponectin and iridine (also known as adipo-mycin) are two circulating adipokines, which have been shown to exert a protective effect against BC, whereas the majority of circulating adipokines, including leptin, resistin, visfatin, osteopontin, apelin, and lipocalin, have been associated with oncogenesis [39]. Adiponectin promotes insulin sensitivity and cell proliferation and acts as a protective factor against tumor progression and exerts its function by binding to the AdipoR1 and AdipoR2 receptors [36,39]. According to a recent epidemiological research, adiponectin and malignancies linked to obesity are inversely connected, and the risk has been linked to low serum levels of adiponectin, whereas high serum levels may exert protective effects against it [39,40]. Contrary to that, leptin is mostly produced in the adipose tissue and through the leptin receptor (LEPR), which is highly expressed in BC cells, it exerts a variety of pre-oncogenic mitogenic effects, and it activates PI3K/AKT and JAK/STAT pathways and induces their proliferation [41,42,43].

## 2. Metformin and Its Mechanisms

**Figure 1:**
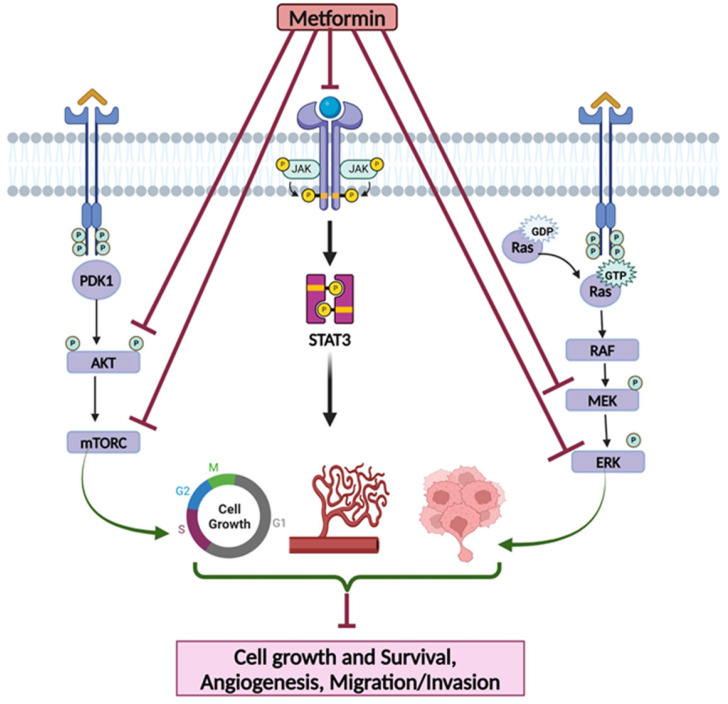
Schematic representation of metformin’s mechanisms of action. Metformin targets PDK1-AKT-mTOR, JAK-STAT3, and RAS-RAF-MAPK signaling cascades leading to the inhibition of cellular activities, including cell growth and survival, migration/invasion, and angiogenesis.

Several anti-diabetics have been used for the treatment of cancer. However, their effectiveness in the long term is often compromised due to the development of tumor resistance and associated side effects [44,45]. To that end, repurposed drugs such as metformin due to their ability to target common mechanisms that favor cancer growth or tumor resistance have been explored in combination with other therapeutic agents for the treatment of BC [46]. Metformin belongs to the Biguanide family, and is one of the most prescribed and the first line agent for treating T2D after lifestyle modifications [44]. Metformin lowers blood glucose levels by decreasing hepatic glucose production and intestinal absorption of glucose and improves insulin sensitivity [47]. Notably, metformin targets several signaling cascades, including the PI3K/AKT/mTOR and RAS/RAF/MAPK pathways. Although metformin-mediated suppression of the PI3K/Akt/mTOR signaling pathway typically involves AMPK-dependent phosphorylation, AMPK activation is not necessary for these effects [48,49]. Importantly, in all of the ER-positive, HER2-normal, or aberrant BC cell lines analyzed, it was demonstrated that metformin inhibits Akt and mTOR pathways, resulting in the inhibition of cellular proliferation, colony formation, and G1 phase cell cycle arrest [48,49]. In TNBC cell lines, it has been demonstrated that metformin-mediated suppression of the PI3K/Akt/mTOR signaling pathway causes the inhibition of cell replication, S phase cell cycle arrest, and apoptosis as well as a decrease in E2F1 and cyclin D1 levels [30,48,49]. Moreover, as TNBC has high levels of STAT3 signaling pathway activation, which in turn promotes cell proliferation, invasion, migration, metastasis, angiogenesis, immune evasion, treatment resistance, and inhibition of apoptosis, metformin is shown to target STAT3 activation (i.e., phosphorylation at both Ser727 and Tyr705 residues) in TNBC [49,50]. The schematic representation of metformin’s mechanisms of action is shown in Figure 1. In this review, we highlight the recent updates on the mechanistic insights and the efficacy of metformin and metformin-based approaches for the treatment of TNBC.

## 3. Implications of Metformin and Metformin-Based Combination Approaches for TNBC

### 3.1. Evidence from In Vitro and In Vivo Studies

Several studies have evaluated the effects of metformin in TNBC using various experimental model systems ranging from in vitro to in vivo studies. Given below, we logically discuss the experimental evidence exploring metformin’s effects on metabolic pathways, and then its combination with other therapeutic agents, targeting cell signaling pathways, approaches to enhance metformin’s effects, and combination with natural anticancer agents.

#### 3.1.1. Metformin’s Effects on Metabolic Pathways

As cellular metabolism is one of the major factors influencing the growth of cancer cells, Amaral and colleagues conducted a series of in vitro experiments to investigate the effect of short- and long-term exposure to metformin on the MDA-MB-231 TNBC cell line [51]. The parameters evaluated were glycemic reuptake, GLUT1 mRNA expression, lactate production, cell viability, proliferation, and culture growth. While the cellular glucose uptake was found to be significantly decreased at short-term exposure (26 min), the long-term exposure to metformin (24 h) revealed a significant increase in glucose reuptake. It is postulated that the inhibition happening at short-term exposure was possibly mediated through direct GLUT1 inhibition. This postulation was further verified by the addition of 2-deoxyglucose (2-DG), a glucose transport protein (GLUT) inhibitor, and a glucose analog. This addition resulted in a significant decrease in glycemic reuptake. Furthermore, as MDA-MB-231 cells undergo aerobic glycolysis, the increased uptake of glucose resulted in an increased formation of lactate as well. In addition, with long-term cell exposure to metformin, cell viability and growth were both significantly reduced in a concentration-dependent manner [51]. Overall, these findings demonstrated that cancer cell exposure to metformin resulted in sensitizing it to glucose, and when sensitized cells were introduced to a glucose reuptake inhibitor, a starvation state was created, leading to apoptosis. A summary of studies highlighting metformin’s role and mechanisms with or without other therapeutic approaches is given in Table 1.

Another study performed by Koker and colleagues aimed to evaluate the effect of metformin’s long-term exposure on the metastatic profile of different BC genomic subtypes [52]. The study examined changes in gene expression using genomically different cell lines, including MDA-MB-231 for Triple-Negative B (TNB), MDA-MB-468 for Triple-Negative A (TNA), and MCF7 for +ER/+PR, which were subjected to a medium containing high glucose and metformin concentrations. After generating metformin-resistant cell lines, they were exposed to metformin for a week. The changes in their transcriptional profiles indicated that ZEB1, vimentin, and MMP9 expressions were upregulated in MDA-MB-468 cells. The findings of increased expression of epithelial–mesenchymal transition (EMT) markers (ZEB1 and vimentin) led to the exploration of other EMT markers. Further investigation revealed that metformin downregulated E-cadherin, claudin, and β-catenin and upregulated N-cadherin, MMP2, Slug and Snail; the findings that support the involvement of EMT in metastasis [52]. Moreover, a cell viability study showed that the combined treatment of metformin and LY294002, a PI3K inhibitor, significantly reduced cell viability as compared to individual treatments. In addition, wound healing assay demonstrated that metformin inhibits cell migration in a concentration-dependent manner. Interestingly, the morphology of MDA-MB-468 cells was changed from the usual spherical shape to a spindle-like appearance [52]. The study concludes that the genomic subtype of BC plays a role in determining whether to include metformin in the treatment plan as it might contribute to metastasis in TNA cells.

On a similar line, studies by Li and colleagues demonstrated that metformin treatment in glucose-deprived MBA-MB-231 cells augments the rate of inhibition of cell proliferation and the induction of apoptosis compared to metformin treatment in glucose-supplemented cells [53]. Notably, RNA-seq analysis led to the identification of the unfolded protein response of endoplasmic reticulum (UPR^ER^). Importantly, metformin in combination with glucose deprivation resulted in the significantly higher activation of UPR and cell apoptosis compared to glucose starvation alone. Mechanistically, this metformin and glucose deprivation-induced effects were found to be mediated by the activation of UPR-related pro-apoptotic genes, such as activating transcription factor-4 (ATF4), ATF3, and C/EBP Homologous Protein (CHOP), and the reduced expression of anti-apoptotic, BCL-2, and BCL-xl signaling pathways compared to glucose deprivation and metformin alone [53]. Interestingly, the depletion of ATP by metformin resulted in an energy crisis, which was enhanced by glucose deprivation, leading to the impairment in the homeostasis of endoplasmic reticulum, causing UPR activation and cell apoptosis by ATF4/ATF3/CHOP signaling [53]. Overall, these studies indicated that the synergy of metformin in the inhibition of cell proliferation is achieved when combined with glucose deprivation.

In addition to its use as a single agent, metformin has also been explored in combination with different chemotherapeutic agents or antimetabolites against TNBC [54]. Studies by Samuel and colleagues investigated the relevance of targeting glucose metabolism as an alternative approach to TNBC treatment and investigated the mechanisms using MDA-MB-231 and MDA-MB-468 TNBC cell lines [54]. The authors used two separate approaches, one where glucose-starved cells were treated with metformin, and the second where cells were treated with a glycolytic inhibitor, 2DG, and their impacts on cellular activities and cell signaling pathways were determined. The data demonstrated that both 2DG and metformin inhibited cell proliferation in a dose-dependent manner, and significantly higher effects were noticed with a combination of 2DG and metformin [54]. Importantly, glucose starvation reduced cell viability, which was enhanced by metformin treatment compared to glucose non-starved cells. However, in glucose-supplemented cells, neither metformin nor 2DG, or their combination, exhibited any effect on cell viability [54]. Similar effects were noticed in apoptosis induction. Interestingly, 2DG alone or the combination of 2DG with metformin significantly induced late apoptosis in MDA-MB-468 cells, but had no effect in MDA-MB-231 cells. Analysis of glycolysis revealed higher basal levels in MDA-MB-231 cells compared to MDA-MB-468 cells, and that metformin treatment increased glycolysis in both these cell lines. While 2DG treatment reduced the rate of glycolysis in MDA-MB-231 (not in MDA-MB-468 cells), metformin increased the rate of glycolysis in 2DG-treated cells compared to 2DG-alone treatment [54]. Mechanistic studies indicated that a combination of metformin and 2DG in glucose-starved cells resulted in the inhibition of activation of the mTOR pathway and its downstream targets, Rap, 4EBP1, and S6, as compared to glucose starvation alone and 2DG treatment alone in both the MDA-MB-231 and MDA-MB-468 cell lines. Overall, these findings indicated that both these cell lines exhibit differential responses to 2DG, metformin, and their combination, which could be due to the metabolic heterogeneity or differences in glucose/glycolysis dependence between the cell types.

#### 3.1.2. Metformin’s Effects in Combination Therapy Efficacy

Rico and colleagues investigated the role of metformin and propranolol metronomic exposure to BC cells in in vitro and in vivo models [55]. In in vitro studies, five different cell lines were used (4T1, M-406, M-23p, MCF-7, and MDA-MB-231) to determine the effects of both the drugs on cell proliferation, clonogenic efficiency, apoptosis, migration, invasion, and metabolic potential. Interestingly, it was found that the drug combination significantly decreased cancer cell proliferation, mitochondrial activity, migration, and invasion. Furthermore, the combination regimen induced apoptosis to a greater extent as compared to individual agents. For the in vivo investigation, female mice underwent tumor implantation with the 4T1 and M-406 cell lines and then randomly divided into three groups: the control group received regular drinking water, the metformin only group received 400 mg/kg BW/day metformin in drinking water, the propranolol only group received 7 mg/kg BW/day in drinking water, and a combination group received both the drugs at given concentrations. At the end of a two-week period, the mice were euthanized, and their lungs were extracted to evaluate metastasis. As cell invasion was significantly reduced in the in vitro analysis, these findings were further validated via in vivo studies demonstrating that the combination treatment resulted in the prevention of tumor metastasis and tumor cell proliferation, as visualized by the Ki67 proliferation marker. As both agents showed no signs of toxicity, either individually or in combination, these findings indicated that the combination therapy could be used as a promising treatment modality for TNBC [55].

Along similar lines, Anselmino and colleagues conducted in vitro and in vivo studies to investigate the outcome of combining metformin with propranolol (M+P) in TNBC [56]. Since M+P combination was found to have ramifications on metastasis, the authors used the 4T1 cell line because of its higher tendency to metastasize and colonize into the lungs. The studies demonstrated that M+P had an effect on different stages of metastasis; intravasation and extravasation. To monitor the effect of M+P on intravasation, 4T1 cells expressing green fluorescent protein (GFP) were injected into BALB mice. Using flow cytometry, the detection of green cells revealed that M+P resulted in a significant decreased circulating tumor cell survival. To assess the extravasation stage, the presence of green cells in the lungs was evaluated using confocal microscopy [56]. After 48 h of the initial injection, the number of malignant cells in treated mice or mice injected with pretreated cells was significantly decreased. The second main finding was that M+P might possibly be an effective preventative strategy after-surgery adjuvant. This postulation was attained as the authors extirpated the tumors and randomized the mice into two post-surgery groups. After that, the mice were euthanized and the reduction in tumor growth with splenomegaly and lung metastasis was observed. However, the reduction was not statistically significant [56]. Overall, this investigation provides the ground to explore M+P’s role as adjuvant therapy in TNBC, especially that it transcends the common disadvantages of chemotherapy, such as high cost and toxicity.

Given that mitochondria remain a significant source of generating adenosine triphosphate (ATP), the main source of energy for various cellular processes, tumor cells also utilize mitochondrial respiration to support their bioenergetic demands. As the reprogramming of mitochondrial fatty acid β-oxidation is a crucial metabolic pathway in TNBC, several studies have demonstrated that TNBC cells exploit the metabolic hybrid state to utilize both glycolysis and oxidative phosphorylation (OSPHOS) pathways for their support [77,78,79]. Thus, approaches to target mitochondrial metabolism and/or modulating the mitochondrial complex I function offer a promising approach for cancer therapy, including TNBC [80,81,82]. Importantly, biguanides such as metformin have been shown to regulate tumor cell activities via mechanisms involving mitochondrial reprogramming in TNBC [71,83].

To that end, a study by Repas and colleagues evaluated the effect of metformin combined with 2-DG in two aspects; mitochondrial biogenesis and protein-programmed death-ligand 1 (PD-L1) expression [57]. Using TNBC cell lines MDA-MB-231 and BT-549, the authors examined the following parameters: mitochondrial mass, total cell number, mitophagy, ATP production, and endoplasmic reticulum stress. To explore the effect on mitochondrial mass, the cell lines were treated with metformin and 2DG alone or their combination. In MDA-MB-231 cells, the only group resulting in a statistically significant finding was the combination of metformin with 2DG, with a 165% increase in mitochondrial mass compared to the control levels. On the other hand, in BT-549 cells, similar trends were observed only upon adapting the cell culture medium to match the one in MDA-MB-231 through the addition of insulin and pyruvate. This increase in mass was found to be associated with increased mitochondrial size, rather than number, as confirmed by mtDNA quantification assay. With regard to the total cell number, a significantly decreased cell number was observed in higher concentration 2DG alone, metformin + 2DG, and metformin + higher concentration 2DG. Although both cell lines responded with a similar trend, BT-549 had a more prominent decrease in cell number. In addition, cells treated with metformin + 2DG maintained their mitophagy, which further confirmed that the increase in mitochondrial mass is related to the increase in number and not to mitophagy. Furthermore, when ATP production was measured by Seahorse Real-Time ATP Production Rate Assay, full suppression was achieved in all treatment groups with a high glucose environment [57]. However, in a standard glucose environment, the suppression of ATP production was achieved only in combination groups rather than single agent ones. Moreover, the combination treatment was found to induce ER stress through the suppression of protein N-glycosylation, as indicated by the concanavalin A staining technique. While the expression of PD-L1 and programmed cell death 1 (PD-1) proteins was unaffected in both cell lines, their levels were found to be suppressed in activated Jurkat cells [57]. In conclusion, the combination strategy could be explored as a potential therapeutic strategy to target the PD-1/PD-L1 axis.

Another in vitro/in vivo study by Tan and colleagues investigated the effect of combining metformin and rapamycin, an mTOR inhibitor, in the treatment of TNBC through inhibiting PD-1 and PD-L1 [58]. The in vitro studies validated that TNBC cell lines have increased PD-L1 mRNA expression. This confirmation was made through comparing RNA-seq data of the MDA-MB-231, MDA-MB-468, and MDA-MB-453 TNBC cell lines with non-TNBC cell lines (MCF-7, T47d, SK-BR-3, and 4T1). It was found that the TNBC cell lines had significantly higher levels of PD-L1 expression. The next studies evaluated which TNBC cell line would be preferred to use in the mice study, and for that the activation of mTOR and p-AMPK levels were evaluated in all cell lines. The choice landed on MDA-MB-231, as it was the only line with low p-AMPK levels. In addition, upon treatment with rapamycin, the downregulation of PD-L1 and p-S6 expression was observed. A similar finding was later confirmed via in vivo studies with a mice xenograft model. It was revealed that mice treated with rapamycin or metformin had a significantly smaller tumor growth as compared to those of the control group, respectively. In addition, while all mice in the control group developed lung metastasis, only two out of four mice in the rapamycin group developed metastasis as compared to three out of four mice in the metformin group. However, those that developed metastasis in the rapamycin and metformin groups had a significantly smaller and lower number of nodules, respectively. Furthermore, the combination of PD-1 monoclonal antibodies (PD-1 McAb) with rapamycin resulted in a more significant suppression of tumor growth compared to rapamycin alone, respectively. Similarly, the combination of PD-1 McAb with metformin led to the same finding [58]. Therefore, it is deduced that the combination of PD-1 McAb with either rapamycin or metformin is synergistic in terms of tumor growth reduction. Moreover, the cotreatment of PD-1 McAb with either rapamycin or with metformin had an additive effect on tumor apoptosis induction, as indicated by the infiltrating number of CD8^+^ T lymphocytes [58]. In summary, the combination may serve as a potential therapeutic strategy due to their ability to block the PD-1/PD-L1 pathway, increasing the number of tumor-infiltrating lymphocytes and decreasing PD-L1 expression.

A study conducted by Xue and colleagues aimed to investigate the role of combining metformin and BMS-754807, an inhibitor of insulin receptor (IR) and insulin-like growth factor 1 receptor (IGF-1R) in TNBC [59]. To assess the role of the combination vs. single drugs, the authors used MTS assays to test cell viability and CompuSyn software to calculate the combination index (CI) to determine whether the effects are synergistic, additive, or antagonistic (CI < 1, =1, and >1, respectively). After that, a reverse phase protein lysate microarray (RPPA) was used to investigate the underlying molecular mechanism leading to synergism. A total of 13 TNBC cell lines, BT-549, HCC1937, HCC38, HCC1806, HCC70, HCC1395, MDA-MB-231, MDA-MB-436, BT-20, MDA-MB-453, Hs 578T, MDA-MB-157, and MDA-MB-468, were used. Interestingly, the CI data indicated that metformin and BMS-754807 had a synergistic effect on 11 out of 13 cell lines. Among the 11 cell lines, the most sensitive lines were BT-20, HCC1806, MDA-MB-436, HCC70, and MDA-MB-468. In addition, as confirmed by RPPA analysis, while metformin alone significantly altered 35 proteins, BMS-754807 significantly altered 118 proteins in HCC1806 cells, among which 19 proteins were common between the two agents [59]. Furthermore, protein analysis revealed a significant depletion of S-phase kinase-associated protein 2 (skp2) levels caused by the combination as compared to single drugs. This depletion resulted in an accumulation of the cell cycle inhibitor protein p27, causing it to be upregulated with increased phosphorylation [59]. In conclusion, the findings of the study pave the way for in vivo preclinical investigation, as the combination is found to be effective in blocking cell growth and proliferation in the majority of TNBC cell lines.

An additional in vitro study conducted by Cheng and colleagues investigated the role of combining resveratrol, a phytochemical with antioxidant and antitumor effects, with metformin [60]. The first studies evaluated the dose–response effect of resveratrol on cell viability using the MDA-MB-231 cell line and demonstrated that low concentrations (LRes, 0–10 μg/mL) induced cell proliferation and high concentrations (HRes, 20–80 μg/mL) resulted in the inhibition of cell growth. The next studies determined the effect of metformin alone and its cotreatment (0–40 mM) with resveratrol. As expected, metformin was effective in reducing cell proliferation in a dose-dependent manner [60]. Furthermore, in combination, metformin inhibited cell proliferation caused by LRes but enhanced the inhibition of cell growth caused by HRes. Moreover, metformin was effective in suppressing cell migration induced by resveratrol. To determine the underlying mechanisms of HRes and LRes and the accompanying synergism with metformin, the authors demonstrated that the JAK3/STAT3 pathway is responsible for the enhanced proliferation and migration in an environment of LRes and metformin plays an effective role in inhibiting its phosphorylation. In an HRes environment, the inhibition of the MAPK pathway was responsible for the resulting apoptosis, which was augmented by metformin. Moreover, HRes upregulated the antioxidants, the superoxide dismutase 3 (SOD3) and prostamide/prostaglandin F synthase (FAM213B) genes, indicating its antioxidative effect [60]. In conclusion, the findings suggest that a combination of metformin with resveratrol could be used to enhance a synergistic treatment modality of TNBC.

Along similar lines, Lee and colleagues studied the effect of metformin combined with cisplatin on TNBC cells in in vitro and in vivo studies [61]. The in vitro investigation comprised evaluating cell viability, proliferation, migration, invasion, and the regulation of the protein-coding gene RAD51 expression on Hs 578T and MDA-MB-231 human TNBC cell lines. It was found that compared to either cisplatin or metformin, the combination of both agents resulted in the significant reduction in cell viability in a dose-dependent manner and exerted a potent antiproliferative effect on TNBC cells [61]. Furthermore, the dual therapy was found to significantly reduce the migration and invasion of cancerous cells as confirmed with wound healing assays. To assess the effect on RAD51 expression, cells were exposed to cisplatin, metformin, and to cisplatin and metformin. The cells exposed to cisplatin only revealed RAD51 upregulation. However, with metformin and in combination with cisplatin, it resulted in the suppression of cisplatin-mediated upregulation of RAD51, associated with its decreased stability. Moreover, the in vitro findings were validated with an in vivo study where the mice were randomly assigned to four groups; control, metformin only, cisplatin only, or cisplatin combined with metformin. At the end of a 21-day period, the mice were euthanized, and weights of their tumors were measured. The combination group had a significantly lower average tumor weight as compared to the metformin and cisplatin alone groups, respectively [61]. Overall, the study concluded that metformin shows promising effects in the treatment of TNBC and exerts synergism when combined with cisplatin.

Another in vitro study by Sahu and colleagues aimed to explore the role of combining metformin with cisplatin and electric pulses (EPs) in terms of cell viability, colony-forming ability, oxidative stress, and glucose consumption [62]. To carry out this experiment, MDA-MB-231 TNBC cells and MCF10A non-cancerous normal mammary cells were used. To deliver EPs, a wave pulse generator was used to deliver eight square wave pulses of 1000 V/cm at 100 μs at one second intervals. In the experiment, cells were treated with (1) EP only; (2) metformin only; (3) varying increasing concentrations of cisplatin only; (4) cisplatin in combination with metformin (cis + met) with EP; and 5) cis + met without EP. At a time point of 24 h, when cells treated with highest concentration cisplatin+ metformin were subjected to EP, the cell viability decreased from 48.4% to 25.86% in MDA-MB-231 cells (i.e., EP caused a statistically significant two-fold decrease in cell viability as compared to that of cisplatin + metformin treatment only). On the contrary, cells treated with EP alone and metformin alone maintained high levels of cell viability at 79.45% and 85.20%, respectively. Conversely, MCF10A cells did not show any statistically significant difference from the control and maintained high percentages of cell viability for all groups. This confirms that normal mammary cells (MCF10A) are not respondent to the treatment of interest, unlike cancerous TNBC cells (MDA-MB-231). In addition, colony-forming assay was performed to assess the cytotoxic effects of the combination on both cell lines. In MDA-MB-231 cells, the colony survival ability decreased as the concentration of the two agents increased, with further reduction upon the addition of EP. Furthermore, oxidative stress was assessed through measurement of ROS. The highest level of ROS was found in cells exposed to EP and treated with 30 μM cisplatin+ metformin. The trend of data showed much higher ROS levels in groups treated with EP + cisplatin + metformin as compared to groups treated with cisplatin + metformin alone. This finding indicates that EP enhances the reuptake of therapeutic agents by the cancerous cells [62]. Moreover, a glucose metabolite assay was used to measure glucose consumption by cancerous cells and it was found that ATP depletion consequently resulted in cell death. Overall, this investigation provides the impetus for further research on the role of electrochemotherapy in TNBC treatment modalities.

In a study conducted by Babak, Chong, et al. [63], metformin and phenformin prodrugs were conjugated with gold (Au) chemical, and the resultant five organometallic novel compounds (i.e., 1–3met, 1phen, and 1met*, where X = Cl for 1met* and X = PF_6_ for 1met) were tested in vitro and in vivo. The novelty behind the formulated prodrugs was meant to serve the purpose of releasing the active compound inside the cancer cell and ascertain the synergism of both pharmacophores. For in vitro studies, the MDA-MB-231 cell line was treated with increasing concentrations of the compounds for 24 h. When the intracellular accumulation of the compounds was assessed, all complexes were shown to accumulate intracellularly in a dose-dependent manner, with 3met having the highest accumulation. Moreover, the findings of increased AMPK phosphorylation and decreased mTOR phosphorylation were similar between the formulated drugs and uncoordinated metformin. After the in vitro studies, the lead compound 3met was selected for the in vivo investigation as it had the highest in vitro activity due to its increased (6000-folds) cytotoxicity, as compared to uncoordinated metformin. Firstly, mice were daily injected with 5, 10, 15, and 20 mg/kg of 3met in order to determine the maximum tolerated dose (MTD). At a dose of 20 mg/kg, mice were found to develop weight loss and kidney and liver toxicity, making 15 mg/kg the MTD. After that, MDA-MB-231 cells were implanted into the mice, followed by injection with 15 mg/kg 3met for 6 weeks. A significant reduction in tumor burden was noted, as compared to the control on week 3, week 4, and week 5. When the biodistribution of Au was assessed in different mice organs, it was found that 3met was accumulated in tumors, since the Au content appeared to be 3–5 fold higher in tumors as compared to the heart, lung, spleen, and kidneys and 3–20 fold higher than that in the brain, liver, and bone. Additionally, H&E staining and the QuPath algorithm were used to assess histological changes and necrosis, respectively. The studies revealed marked lymphohistiocytic infiltration in 3met-treated mice, a finding suggestive of an enhanced immune response to the tumor and linked to enhanced drug sensitivity and disease prognosis. In terms of necrosis, 3met-treated mice underwent more necrosis (33 ± 4%) as compared to vehicle-treated mice (10 ± 1%), which indicates that 3met exerts an anticancer effect [63]. Overall, the study reports suggest that Au and metformin pharmacophores are successful in abrogating cancerous mitochondrial respiration, leading to cell death.

Often, the efficacy of anti-cancer drugs is compromised via several factors. The reduced efficacy of metformin hydrocholoride is correlated with its hydrophilicity and poor permeability. To that end, studies by Saeed and colleagues exploited an alternative approach to enhance the efficacy of metformin via synthesizing lipophilic salts of metformin that contain a bulky chain of anionic permeation enhancers to increase the lipophilicity and enhance permeation [64]. Of several lipophilic permeation enhancers, metformin docusate was selected due to its improved activity, including lipid solubility. Notably, substantially higher anticancer activity of metformin docusate was observed in both drug-sensitive MYCN-2 and drug-resistant SK-N-Be2c neuroblastoma cell lines as well as in HepG2 hepatocellular carcinoma, and MDA-MB-231 TNBC as compared to metformin hydrocholoride, and sodium docusate [64]. Overall, these studies indicated that metformin docusate exerts greater anticancer effects due to its enhanced lipophilicity and lipid solubility.

As patients with TNBC treated with cisplatin have been reported to have hypoxia and an increased cancer stem cell (CSC) population, Sulaiman and colleagues conducted a study that aimed to explore the role of metformin combined with gefitinib in overcoming cisplatin resistance in TNBC [65]. In this in vitro investigation, the authors explored the effect of the combination of cisplatin, metformin, and gefitinib (CMG) on cell viability, hypoxia gene expression, and CSC population, namely CD44^+^/CD24^−^ and aldehyde dehydrogenase (ALDH+) activity. To assess the effect of CMG on cell viability, MDA-MB-231 and SUM 149-PT cell lines were treated with CMG for 120 h. Both cell lines were found to have a significant reduction in cell viability in the CMG group as compared to individual or two-agent combination. To investigate whether CMG suppresses hypoxia, the hypoxia-inducible factor (HIF) was studied via luciferase activity. As the HIF activity in cells treated with cisplatin alone was increased, it was reduced in cells treated with metformin + gefitinib. Remarkably, HIF activity was antagonized in cells treated with CMG. Additionally, upon examining CSCs, CMG was found to inhibit CD44^+^/CD24^−^ by ~90%. However, ALDH+ was inhibited by ~90% by the combination of cisplatin + gefitinib, metformin + gefitinib, and CMG in both cell lines [65]. In conclusion, the findings of the study suggested a possible clinical benefit of CMG through attenuating cisplatin-induced drug resistance and, therefore, enhancing its efficacy in TNBC treatment.

In another in vitro study, a microarray analysis was used to explore the differential expression of genes in the MCF-7 and MDA-MB-231 BC cell lines when subjected to glucose starvation (GS) or glucose-lowering agents such as 2-DG or metformin [66]. To fulfill this aim, both the cell lines were treated for 48 h with either (1) metformin or (2) 2-DG, and then (3) placed in a GS environment by decreasing the glucose concentration stepwise to zero, followed by keeping the cells in FBS only for 1 week. After that, gene profiling was conducted via Affymetrix Human Clariom S microarrays. The following findings were recorded in MDA-MB-231 cells: (1) unfolded protein response upregulation (chaperone activation) was detected as a sign of endoplasmic reticulum stress; (2) clusters related to apoptotic processes were upregulated; (3) DNA replication was inhibited. Additionally, after treatment with 2-DG, autophagy was induced. Among the three groups, a downregulated cluster of DNA methylation (polo-like kinase 1 activity) was detected only in cells subjected to GS. In addition, in the GS and metformin treatment groups, cellular response to reactive oxygen species was upregulated, but clusters related to DNA repair mechanisms were downregulated. Notably, MDA-MB-231, a TNBC cell line, was more responsive to all three interventions as compared to MCF-7, a non-TNBC cell line [66]. To conclude, the GS environment was found to be the most effective in damaging cancerous cells as compared to 2-DG and metformin. Thus, combining GS with metformin could potentially render chemotherapeutic outcomes more successful.

An in vitro study conducted by Liu and colleagues aimed to investigate whether metformin enhances the expression of TNF-related apoptosis-inducing ligand (TRAIL) in TNBC and non-small cell lung cancer (NSCLC) [67]. Another objective was to assess whether TRAIL-induced apoptosis plays a role in the antitumor activity exerted by metformin. To that end, cell viability, TRAIL expression, and the effect of blocking TRAIL expression were evaluated in vitro using HCC70, MDA-MB-468, and BT549 cell lines. The cell viability was assessed with metformin using HCC70, MDA-MB-468, and BT549 cell lines for 48 h. After that, the analysis of metformin-mediated apoptosis was evaluated by ELISA assay, which revealed that metformin significantly inhibited cell viability through inducing apoptosis in a dose-dependent manner. Western blot analysis further confirmed this finding as enhanced PARP cleavage and elevated levels of active caspase-8 and caspase-3 were noticed. In addition, the apoptotic process was found to be triggered by increased expression of TRAIL. To further validate the finding, the investigators attempted to block TRAIL with the recombinant TRAIL- R2-Fc chimera protein. Interestingly, the inhibition of TRAIL led to a significant attenuation of metformin-induced apoptosis in TNBC [67]. Overall, this study identifies the other potential mechanism of metformin as an antitumoral agent.

Cai and colleagues performed an in vivo study that aims to investigate the efficacious dose of metformin and its relationship to metformin transporters named organic cation transporter 3 (OCT3) [68]. The study was designed to test ER+, PR+ (MCF-7 cell line), or TNBC (MDA-MB-468 cell line) breast tumors in female athymic mice followed by randomizing them to different groups. Since MDA-MB-468 cells express OCT3 at considerably higher levels as compared to that of MCF-7 cells, the investigator used an OCT3 gene vector to generate OCT3-overexpressing MCF7 (i.e., OCT-MCF7) cells. The randomized groups were those receiving either (1) saline; (2) 30 mg/kg/week paclitaxel for MCF-7 tumors or 50 mg/kg/week carboplatin for MDA-MB-468 tumors; (3) 360 mg/kg/day metformin alone; and (4) 30 mg/kg/week paclitaxel or 50 mg/kg/week carboplatin plus metformin at 12, 36, 120, and 360 mg/kg/day. After that, metformin concentrations were measured through collecting blood samples from the mice at different hours. At the end of a 20-day period, mice were euthanized, and the tumors were collected for analysis. The results indicated that metformin was more efficacious in OCT3-MCF7 cells as compared to MCF-7 cells. Furthermore, to achieve a similar intratumoral exposure of metformin in MDA-MB-468 cells, triple the dose of metformin has to be used in MCF-7 tumors. The study concluded that the expression of the transporter is a major determinant of metformin anticancer efficacy and a higher dose maybe required to achieve beneficial effects [68].

With the increasing body of evidence growing around the use of metformin and its combination with standard antitumoral agents, Pateliya and colleagues [69] investigated the effect of naringenin to evaluate the efficacy and safety of combining naringenin, a flavonoid, and metformin as adjuncts to doxorubicin chemotherapy in the scope of TNBC treatment in vitro and in vivo. The in vitro part of the study used MDA-MB-231 and 4T1 cell lines to evaluate cell viability. To that end, the MDA-MB-231 cells were subjected to various treatments: (1) doxorubicin alone; (2) doxorubicin + naringenin; (3) doxorubicin + metformin; and (4) doxorubicin + naringenin + metformin. The cotreatment with metformin and doxorubicin did not result in any significant difference as compared to doxorubicin alone. On the other hand, when MDA-MB-231 cells were treated with doxorubicin + naringenin, they were significantly more sensitive to doxorubicin, and resulted in a significant decrease in cell viability. Additionally, the cotreatment with both naringenin + metformin resulted in a significant increased efficacy (i.e., decrease in cell viability). However, in the 4T1 cell line, the individual treatment of metformin or naringenin with doxorubicin did not result in any significant improvement. On the contrary, when both metformin and naringenin were used in combination, they showed a significant reduction in cellular viability. Consistently, a significant reduction in tumor volume was found in mice injected with MDA-MB-231 cells. Furthermore, mice treated with doxorubicin 3 mg/kg + metformin 100 mg/kg + naringenin 50 mg/kg were significantly lower in TNF-α as compared to those treated with doxorubicin alone, signifying a reduction in pro-inflammatory cytokines. In addition, the combination was able to achieve a significant reduction in Ki-67 expression, which implies favorable results in terms of proliferation inhibition [69]. Therefore, the findings suggested that naringenin and metformin could be helpful in enhancing the sensitivity of cancer cells to chemotherapy. However, more in-depth human clinical trials are warranted.

#### 3.1.3. Metformin’s Effects on Targeting Cell Signaling Pathways

A study conducted by Strekalova and colleagues aimed to investigate the role of metformin when combined with TRAIL receptor agonists, namely Mapatumumab, Lexatumumab, and recombinant TRAIL peptide [70]. To answer this question, human TNBC cell lines, MDA-MB-231-mCherry, GILM2, and MDA-MB-468 were utilized in vitro and NOD scid IL2 receptor γ chain knockout (NSG) mice were used in vivo. In in vitro studies, the authors assessed the impact of metformin on four aspects: TRAIL receptor sensitization to agonists, apoptosis induction, receptor expression over cell surface, and X-linked inhibitor of apoptosis protein (XIAP) expression. When examining the effect of metformin on TRAIL receptor agonists, it was found to sensitize the three cell lines at a minimum metformin concentration of 0.5 mM. Notably, the most sensitized cell line was MDA-MB-231-mCherry followed by GILM2. To determine whether metformin enhanced apoptosis induction by examining caspase activation, it was found that metformin augmented the TRAIL-induced caspase activation in all three cell lines. To determine whether metformin could also increase the expression of TRAIL-R1 and TRAIL-R2 receptors on the cell surface, it was found that both receptor expressions were unaffected by metformin. On the other hand, real-time PCR results showed only a slight increase in TRAIL-R2 mRNA levels in one cell line, MDA- MB-468. Therefore, the overall finding is that metformin does not alter the cell surface expression of TRAIL receptors on TNBC cells. In addition, metformin was found to downregulate the expression of XIAP. Furthermore, silencing XIAP improved the cell viability of the MDA-MB-231-mCherry cell line. In the in vivo study, the investigators randomized mice into four treatment groups: daily PBS vehicle, 2 mg/mL metformin in drinking water, daily 10 mg/kg TRAIL, and a combination of metformin and TRAIL. After that, the tumor volumes were calculated, and lung metastases were visualized by fluorescence microscopy. Interestingly, the findings of the in vitro investigation were consistent with the in vivo studies, as the combination group had reduced XIAP protein levels. Moreover, the combination treatment inhibited lung metastases to a similar degree of that in TRAIL alone. In a nutshell, the studies indicated that since TNBC still lacks efficacious targeted therapies with little established benefit of TRAIL receptor agonists, the combination of metformin with TRAIL agonists could have promising results [70].

One study by Lee and colleagues investigated the role of metformin combined with hemin as a novel strategy to target BTB and CNC homology 1 (BACH1) in TNBC [71]. As BACH1 has been found to be more expressed in TNBC, the authors carried out an in vitro and in vivo evaluation to determine whether BACH1 could be exploited in sensitizing TNBC cells to metformin. In in vitro studies, human BC cell lines (MDA-MB-436, MDA-MB-468), nonmalignant mammary epithelial cells (MCF10A and 184A1), and BM1 cells were used. The investigators studied the effect of BACH1 depletion on metabolism, the association between BACH1 level and metformin response, the effect of other electron transport chain (ETC) genes in BACH1-depleted cells on metformin resistance, and the effect of introducing hemin to metformin-treated cells. To determine the effect of BACH1 depletion on metabolism, the extracellular acidification rate (ECAR) and oxygen consumption rate (OCR) were measured [71]. The data demonstrated a notable increase in OCR but decreased levels of ECAR in BACH1-depleted cells. This indicated that the loss of BACH1 enhances aerobic respiration in TNBC cells. This was further confirmed by the mass spectrometry analysis, which revealed increased ATP levels and decreased intermediate compounds of the glycolysis pathway. Since the loss of BACH1 increased metabolism, the authors investigated whether this phenomenon could be useful in terms of metformin’s effect on TNBC cells. With the loss of BACH1, metformin was found to be more effective against TNBC cells as it reduced cellular growth and viability. In addition, when pyruvate was added to BACH1-depleted cells, reduced levels of other mitochondrial ETC genes such as COX15 and UQCRC1 were documented. This resulted in metformin resistance, which was mediated through the restoration of TNBC cell growth. Furthermore, as hemin is known to induce BACH1 degradation, the loss of BACH1 was evaluated through treating the BM1 and MB436 cells with hemin. The treatment resulted in a maximal increase in OCR but decreased ECAR. Additionally, hemin reduced cellular growth and viability. This indicates that hemin further enhances the aerobic respiration process in TNBC cells in the presence of metformin.

In in vivo studies, BACH1-depleted MB436 tumor xenografts were treated with or without metformin in athymic nude mice. No change in tumor size in either BACH1-depleted or metformin alone was observed as compared to the control. However, metformin suppressed the growth of BACH1-expressing BM1 or MB436 tumor xenografts. The combination of metformin and hemin resulted in the significant suppression of BM1 and MB436 tumor growth [71]. This highlighted the finding of the hemin sensitization of TNBC cells to metformin. Therefore, the findings suggest that hemin combined with metformin could serve as an effective therapy in improving TNBC treatment outcomes, depending on the expression of the BACH1 gene.

In a study conducted by Wang and colleagues [72], the implication of metformin combination with the C-Jun N-terminal kinase (JNK) signal pathway on functional and exhausted tumor-infiltrated lymphocytes (TILs) was evaluated in TNBC. In the study, 4T1 cells harboring a different JNK expression status were used: Wildtype 4T1 cells having endogenous JNK expression, cells with JNK knock-down (4T1 JNK KD), and cells with overexpressed JNK (4T1 OVE JNK). The analysis of cell viability demonstrated that when metformin was used in a low concentration (0.1–5.0 mmol/L), it had no significant effect in terms of cell viability inhibition. However, higher concentrations were found to be significantly suppressive (10.0 mmol/L and 20.0 mmol/L, respectively). Additionally, cells treated with metformin had a significantly higher JNK expression as compared to cells treated with vehicle control. In addition, the in vivo studies conducted in BALB/c mice inoculated with wildtype 4T1 and treated with metformin witnessed an increase in CD4^+^ and CD8^+^ TIL cell counts. With respect to CD4^+^ TILs, the count in the metformin group was an 82/high power field (HPF) as compared to 41/HPF in the control. For CD8^+^ TILs, the count increased significantly from 47/HPF in the control to 87/HPF in the metformin group. On the other hand, mice inoculated with 4T1 JNK KD initially had lower (30/HPF) CD4^+^ TILs and CD8^+^ TILs. However, the administration of metformin significantly restored their levels to 62/HPF and 63/HPF, respectively. Furthermore, mice inoculated with wild-type 4T1 cells and treated with metformin showed a significant reduction in the IL-6 levels. However, mice inoculated with JNK KD and OVE JNK did not show a significant change in IL-6 level. Likewise, mice inoculated with wild-type 4T1 had a significant decrease in TNF-α level, whereas mice inoculated with JNK KD and OVE JNK did not show a significant change [72]. In summary, the effects imposed by metformin in terms of the reduction in CD4^+^ and CD8^+^ TILs, IL-6, and TNF-α could potentially serve a role in combined immunotherapy in TNBC.

Another study conducted by Bhardwaj and colleagues aimed to identify a possible prevention-based approach for TNBC [73]. In the in vitro studies, the investigators attempted to identify molecular RNA markers that could possibly be targeted. To that end, an early-stage TNBC development model called an MCF10A-based model is utilized. This model system includes a normal cell line [MCF10A (P)], cellular atypia cell line (MCF10.AT1), in-situ ductal carcinoma cell line (MCF10.DCIS), and an invasive cancer cell line (MCF10.Ca1d). Within this system, the authors employed RNA extraction and qPCR to study the expression of miR-140-3p, an miRNA that is regulated through a cholesterol biosynthesis/mevalonic acid (MVA) pathway. It was found that during the preneoplastic progression, particularly from normal to atypia (MCF10A (P) to MCF10.AT1), miR-140-3p-1 was expressed at ~13–17-fold higher levels than canonical miR-140-3p-2. After that, it is lost during BC progression. Notably, the ectopic expression of miR-140-3p-1 to preneoplastic cells (MCF10.AT1) reduced cell colonizing ability and inhibited cell proliferation. This piece of understanding provided the rationale for the usage of statins in targeting the MVA pathway as a BC preventive measure. This was further confirmed in the study as fluvastatin was found to inhibit cell proliferation in a dose-dependent manner.

To test translate this in an in vitro finding to an in vivo setting, mice were randomized into treatment and control groups where the control group received plain drinking water and the treatment group received fluvastatin 10 mg/kg/day in drinking water. At the end of a 16-week period, mice were euthanized, and tissues were collected. Although the lesions in mice treated with statin were 25% smaller compared to the control, fluvastatin did not inhibit the neoplastic progression. This might be attributed to the fact that along the usage of statins, a chemo-preventive feedback loop is created. This loop could be overruled through activating the AMP-activated protein with metformin or aspirin. On the premises of this understanding, statin-resistant cells (MCF10.AT1-R) were generated and treated with a combination of fluvastatin with aspirin alone, with metformin alone, and with metformin + aspirin for 48 h. The synergistic combination of fluvastatin, metformin, and aspirin resulted in 100% inhibition of cell colonization [73]. Overall, the results of the study suggest that the combination of the three agents together is promising in terms of TNBC prevention.

In another report, Xu and colleagues explored the role of metformin on YAP/TAZ axis regulation and its relation to achieving BC therapeutic effects. To that end, human data were collected followed by in vitro/in vivo investigation [74]. A sample of BC was collected from 80 women who underwent surgical resection, out of which 40 samples of TNBC cases were selected. When the samples were screened for YAP, 30 out of 40 samples were YAP positive (75%) as compared to only 12 out of 40 (30%) in the normal tissue of adjacent breast. Regarding the expression of TAZ, 28 out of 40 samples were TAZ positive (70%) as compared to only 14 out of 40 (35%) in the normal tissue of adjacent breast. This indicated that the expression of YAP/TAZ was significantly higher in TNBC. In addition, the positive expression of YAP/TAZ appeared to significantly correlate with tumor size. In the in vitro investigation, cell viability, migration, growth, and effect on YAP/TAZ expression were evaluated following metformin treatment in the MDA-MB-231 cell line. The results suggested that metformin impeded cell growth in a dose-dependent manner. In addition, metformin induced an apoptosis effect, as demonstrated by a significantly increased cell population in the G1 cell cycle phase. Additionally, the expression of genes encoding YAP/TAZ as well as the mRNA expression of their downstream genes EGFR, CTGF, and CYR61 were significantly decreased, which indicates that metformin hinders the translocation of YAP/TAZ. Furthermore, metformin had a lowering effect on YAP-induced EMT as it decreased the expression of vimentin and N-cadherin and increased E-cadherin [74]. In in vivo studies, mice inoculated with 4T1 cells and treated with metformin did not affect any significant weight loss. However, tumor growth was reduced, as indicated by the significant reduction in tumor volume and weight. In addition, the relative protein levels of YAP/TAZ in tumor tissues of mice treated with metformin were significantly less, as compared to the control [74]. This indicates that the inhibition of tumor growth by metformin could be through hindering the tumorigenic activity of YAP/TAZ.

#### 3.1.4. Approaches to Enhance Metformin’s Effects via Its Delivery Through Nanoparticles

A study published by Basu and colleagues [75] investigated the mechanism of efficacy of hyaluronic acid-engrafted metformin-loaded graphene oxide (HA-GO-Met) nanoparticles as anticancer agents in TNBC in vitro and in vivo. The MDAMB-231 cell line was used in the in vitro investigation to evaluate cell viability and cell migration. The data demonstrated that HA-GO-Met was effective in inducing cell death as compared to GO-Met or metformin alone. In addition, the involvement of miR-10b, a microRNA that has been established to contribute to BC virulence and be correlated with the tumor-suppressing gene PTEN, was studied. As PTEN gets upregulated, miR-10b gets downregulated, forming a mir10b/PTEN axis. The immunocytochemistry and Western blots revealed that cells treated with HA-GO-Met nanoparticles resulted in a downregulation of NFĸB-p65 expression in the nuclear area, but upregulation in the cytosolic region. In control cells, the opposite was observed: upregulation in the nuclear area and downregulation in the cytosolic region. This indicates that HA-GO-Met nanoparticles target the mir10b/PTEN axis. In addition, HA-GO-Met nanoparticles were found to attenuate cell migration, as indicated by bidirectional wound healing assay. In addition, miR-10b inhibited cell migration, which was attributed to the upregulation of PTEN and, consequently, the downregulation of integrinβ1/pFAK signaling. Furthermore, E-cadherin was found to be slightly increased in treated cells, which signifies that HA-GO-Met inhibits EMT transition. Moreover, HA-GO-Met nanoparticles were also tested in vivo through injecting BALB/c mice with 4T1 TNBC cells to induce tumors. After a week, mice were injected with 8 mg/kg of HA-GO-Met nanoparticles intraperitoneally and intravenously. Upon histological analysis of tumor cells, it was revealed that treated mice had a decrease in cancerous proliferative capacity, as indicated by the reduction in nuclear to cytoplasmic area. Furthermore, no evidence of metastasis was found in treated mice, contradictory to controls [75]. Overall, these findings indicate that HA-GO-Met possess promising anti-cancer properties and thus, could be explored with other signaling pathway inhibitors.

In another study by Zhang and colleagues, the role of encapsulating relaxin (RLN), an antifibrotic known to inhibit abnormal fibroblast proliferation, with metformin was investigated in altering the immune microenvironment of tumors in TNBC by reducing cancer-associated fibroblasts (CAFs) [76]. Because achieving a local RLN expression in a tumor was difficult due to several factors, including its small size, and short half-life and vasodilation as a systemic side effect, RLN plasmids (pRLNs) were formulated as a complex with Polymeric metformin (PolyMet) to make PolyMet-pRLN. After that, for enhanced stability, the complex was further developed into a novel nanoparticle lipid poly-γ-glutamic acid (PGA)/PolyMet-pRLN (LPPR) nanoparticle, which serves the purpose of delivering pRLN to the targeted tumor due to its ability to express locally in the target of interest. After formulating the complex, it was tested in vitro and in vivo. As the 4T1 cell line was incubated with LPP and LPPR for 48 h, it was mixed with CAFs evenly at a ratio of 1:1. To observe morphological changes in the tumor spheres, it was daily photographed under a fluorescent microscope. The confocal laser scanning microscopy imaging revealed that coumarins with LPP remained on the edges of the tumor sphere, while those with LPPR were efficient in penetrating deep into the tumor, which indicates the efficiency of pRLN in increasing penetration. Moreover, since CAFs secrete tumor proliferative factors such as anti-alpha smooth muscle actin and fibroblast activation protein alpha (FAPα), and growth factor TGF-β, their levels were evaluated. To assess their levels, cells were treated with either LPPR alone, LPPR + anti-PD-L1, or PBS. Both the LPPR alone and LPPR + anti-PD-L1 group resulted in a reduction in all three factors. However, LPPR + anti-PD-L1 resulted in a greater reduction in the levels of α-SMA (>50%), FAP (>40%), and TGF-β (>30%) compared to PBS. These findings clearly indicated the role of LPPR in aiding anti-PD-L1 therapy success by damaging CAFs. When tested on BALB/c mice, the in vivo findings complemented in vitro studies in that LPPR could aid anti-PD-L1 to overcome its resistance by damaging CAFs and improving anti-tumor immunity, as indicated by increased cytotoxic T cell (i.e., CD3^+^, CD8^+^ and CD4^+^) infiltration to the tumor. After evaluating efficacy, safety investigation revealed no notable weight loss, no ALT, AST, BUN, or CREA abnormality, and no histological damage in the heart, liver, lungs, spleen, and kidneys was detected in H & E staining [76]. The authors conclude that LPPR could have an effective role in enhancing the therapeutic efficiency of anti-PD-L1.

### 3.2. Evidence from Clinical Studies

After the emergence of a wide body of evidence from in vitro and in vivo studies, metformin has been studied in human subjects to evaluate its role in TNBC. Multiple studies have explored the effects of metformin alone or in combination in TNBC patients.

As metformin has been found to exert a crucial role in overcoming the resistance to EGFR inhibition, Fenn and colleagues [84] performed a phase I study with the aim of investigating the MTD of metformin when combined with 150 mg of erlotinib in patients with mTNBC. After determining the MTD of metformin, the authors aimed to explore the clinical benefits as defined by either partial response or stable disease. While the primary end point in the investigation was dose-limiting toxicity (DLT), the secondary end points were the response rate, stable disease rate, and progression-free survival (PFS). In a treatment cycle of 21-day continuous treatment, a total of eight participants underwent one of two metformin treatment regimens: 850 mg twice daily (three patients) and 850 mg trice daily (five patients), with an escalation plan in accordance with a 3+3 design. However, the best response achieved was stable disease in two participants (25%) out of eight patients enrolled. The median progression-free survival was 60 days. Of the two patients who achieved disease stability, the first developed bone metastasis after 79 days, and the second withdrew after 57 days upon developing a skin rash consistent with a hypersensitivity reaction. However, it was unclear whether the reaction was induced by erlotinib or metformin. While the combination was well tolerated with no unexpected adverse effects, due to the absence of a sufficient beneficial clinical effect of this combination, further investigation is warranted [84]. The limitations of this study include the enrollment of a small number of TNBC patients and the best outcomes were instances of stable disease with a median progression-free interval of only 60 days. These data suggest that metformin-based strategies, even in combination, may offer limited benefits when targeting cancer purely from a genetic perspective. A summary of clinical studies is highlighted in Table 2.

In a single randomized study conducted by Patterson and colleagues, the effects of metformin on biomarkers linked to BC prognosis were studied [85]. Those biomarkers were the changes in fasting insulin, glucose, C-reactive protein (CRP), estradiol, testosterone, and sex-hormone binding globulin (SHBG). The investigators enrolled a total of 313 overweight/obese postmenopausal BC survivors (BMI ≥ 25.0 kg/m^2^), 60 of whom were TNBC survivors, for a duration of six months. Additionally, the study was designed to follow a 2 × 2 factorial design and the subjects were randomly assigned to receive metformin, with a gradual increase in dose of up to 1500 mg/day, or placebo. Interestingly, fasting insulin, estradiol, testosterone, and SHBG concentrations were all improved in the metformin arm with statistical significance. Conversely, both glucose and CRP concentrations were not significantly affected by metformin as compared to placebo, and respectively. Given that a high adherence was defined as ≥80% pills taken, even with a reported lower adherence rate in the metformin group (65.9%) as compared to placebo (81.3%, *p* = 0.002), the results were still impactful. The metformin arm had a significantly higher mean weight loss as compared to that of the placebo. Overall, the authors infer from their findings that mildly reduced ER levels caused by metformin and weight loss are advantageous in reducing BC recurrence and its associated mortality [85]. While the risk of developing BC has been linked to type II diabetes and antidiabetic metformin, the exact molecular subtype associated with them was still unrecognized.

Chen and colleagues [86] conducted a multi-center, retrospective case–case clinical study in a population of women aged between 20 and 69 years [86]. The study subjects were a total of 4340 BC diabetic women, of whom 1,992 were ER+/HER2^−^, 324 were ER+/HER2+, 1446 were TNBC, and 578 HER2 were overexpressing (ER-/PR-/HER2+). To draw statistical insights, the authors used a polytomous logistic regression to estimate OR and their corresponding CI. The analysis revealed that patients with type II diabetes had 38% increased odds of TNBC (95% CI, 1.01–1.89) as compared to subjects with ER+/HER2^−^ BC cases. Moreover, the odds of developing H2E BC were observed to be increased among patients with type II diabetics (OR 1.38; 95% CI, 0.93–2.06). However, this estimate was within the limits of (OR 1.41; 95% CI, 1.03–1.95), relative to nondiabetic women. Furthermore, the longer the duration of metformin use before BC diagnosis, the higher the odds were for developing TNBC. Notably, the authors reported that the majority of the study population consisted of White non-Hispanic subjects, which could be a limiting factor in terms of the generalizability of the data. Overall, it is concluded that this study could serve as an epidemiological speculatory tool, given the data could help in identifying populations at increased risk of developing TNBC, such as diabetic women with metformin use [86].

Another large-scale prospective study was performed by Park and colleagues with the purpose of examining the relationship between T2D, the use of metformin, and BC risk prospectively [87]. Between 2003 and 2009, a total of 44,541 subjects aged between 35 and 74 years old with no former diagnosis of BC were enrolled. The investigation, commonly referred to as the sister study, targeted women who have sisters or half-sisters diagnosed with BC. For statistical analysis, the authors used Multivariable Cox proportional hazard models to estimate hazard ratios and 95% confidence intervals. Throughout a median of 8.6 years of follow up, 2678 were diagnosed with BC at least one year after enrollment. The results indicated that patients with T2D maintained on diabetes were not generally found to be at higher risk of BC (HR 0.99; 95% CI, 0.87–1.13). However, subtype analysis revealed otherwise. T2D patients treated with metformin were found to have an increased risk of TNBC (HR 1.40; 95% CI, 0.90–2.16). On the other hand, when patients without T2D were compared with those with T2D maintained on metformin, the use of the drug did not seem to generally contribute to the risk of BC (HR 0.98; 95% CI, 0.83–1.15), but it was associated with a (1) decreased risk of ER+ BC (HR 0.86; 95% CI 0.70–1.05); (2) increased risk of ER-BC (HR 1.25; 95% CI, 0.84–1.88); and (3) increased risk of TNBC (HR 1.74; 95% CI, 1.06–2.83). In addition, the study conductors performed sensitivity analysis and their overall conclusions were unaltered. Therefore, it is concluded that the association of T2D with BC differs depending on the molecular subtype of BC [87].

In addition to these published reports, several clinical trials have been assessing the efficacy of metformin with or without other agents in breast cancer, including TNBC patients [88,89,90,91,92]. A list of ongoing and recently completed clinical trials of metformin use in TNBC is summarized in Table 3.

## 4. Conclusions and Future Perspectives

Multiple experimental studies have confirmed the potential anti-cancer activity of metformin against TNBC, which is attributed to its ability to target various activities of tumor cells, including proliferation, migration, invasion, and angiogenesis, leading to decreased tumor growth and metastasis. Notably, metformin has also been used to overcome drug resistance mechanisms. In addition, several therapeutic agents have been evaluated with metformin and found to exert synergistic antitumor effects. Moreover, metformin can be used as a promising combination approach to target other potential signaling cascades involved in tumor development and/or impede the efficacy of therapeutic agents. While metformin in combination with other therapeutic agents resulted in synergistic antitumor effects in experimental in vitro and in vivo models, it did not show preferable outcomes in clinical settings in patients who have T2D, which remain the ongoing challenges and limitations to be explored in future research. Thus, the identification of potential biomarkers and their implications in selecting a subgroup of TNBC patients who could benefit from metformin treatment is important to determine the best combination approaches, including strategies to avoid adverse events. Taken together, metformin should be evaluated as a novel therapeutic strategy with careful consideration with other anti-cancer agents in clinical trials for the treatment of TNBC. The ability of metformin to target multiple cell-signaling pathways provides a rationale for its exploration with other therapeutic agents for the treatment of TNBC.

## Figures and Tables

**Table 1:** Summary of the in vitro and in vivo studies defining the role and mechanisms of metformin and metformin-based combination approaches in TNBC.

Agents	Target (s)	Parameters	Findings	Refs.
Metformin	GLUT1 mRNA expression	Glycemic reuptake, lactate production, cell viability, and culture growth	Glucose uptake decreased after short-term exposure but increased in long-term exposure. Increased formation of lactate was observed. In addition, the cell viability and growth were reduced in a concentration-dependent manner.	[51]
Metformin	Expressions of ZEB1, Vimentin MMP9, N-cadherin, MMP2, Slug and Snail	Long-term exposure on the metastatic profile of different BC genomic subtypes	Upregulation of ZEB1, Vimentin, MMP9, N-cadherin, MMP2, Slug and Snail expression, and downregulation of E-cadherin, claudin, and β-catenin were noted by metformin treatment. In addition, combined treatment of metformin and LY294002 reduced cell viability as compared to either one of them alone.	[52]
Metformin	UPR	Rate of inhibition of cell proliferation and induction of apoptosis	Metformin + glucose deprivation resulted in higher activation of UPR and cell apoptosis compared to glucose starvation alone.	[53]
Metformin and 2-DG	mTOR	Effects on cellular activities and cell signaling pathways	Metformin and 2DG alone inhibited cell proliferation in a dose-dependent manner and significantly higher effects were noticed with combination. Also, the combination in glucose-starved cells resulted in the inhibition of activation of the mTOR pathway and its downstream targets.	[54]
Metformin and propranolol		Cell proliferation, clonogenic efficiency, apoptosis, migration, invasion and metabolic potential, and in vivo studies	Drug combination reduced cell proliferation, mitochondrial activity, migration, and invasion, and induced apoptosis. In vivo results validated the reduction in invasion and inhibition of cell proliferation.	[55]
Metformin and propranolol		Metastasis, intravasation, and extravasation	The combination of metformin and propranolol resulted in a significant decline in circulating tumor cell survival.	[56]
Metformin and 2DG	PD-L1	Mitochondrial biogenesis and protein PD-L1 expression	Metformin + 2DG resulted in a significant increase in mitochondrial mass, and decrease in PD-L1 expression.	[57]
Metformin and rapamycin	PD-L1 and p-S6	PD-1 and PD-L1 inhibition. In vivo studies.	Rapamycin resulted in the downregulation of PD-L1 and p-S6 expression, and mice treated with rapamycin or metformin had a smaller tumor size as compared to those of the control group.	[58]
Metformin and BMS-754807		Cell viability, combination index	Metformin and BMS-754807 had a synergistic effect on 11 out of 13 cell lines.	[59]
Metformin and resveratrol		Cell viability	Metformin in combination with resveratrol inhibited cell proliferation caused by LRes but enhanced the inhibition of cell growth caused by HRes.	[60]
Metformin and cisplatin		Cell viability, proliferation, migration, invasion, and regulation of RAD51 expression. In vivo measurement of mice tumor.	Cisplatin + metformin resulted in reduced cell viability, cell migration, and invasion and exerted a potent antiproliferative effect on cancer cells. The combination resulted in a lower average tumor weight as compared to metformin and cisplatin alone groups, respectively.	[61]
Metformin, cisplatin, and electric pulses		Cell viability, colony-forming ability, oxidative stress, and glucose consumption	Cell viability and colony forming ability were decreased with cisplatin+ metformin subjected to Eps. When cells were treated with metformin and exposed to EP, glucose levels were decreased and ROS level was increased.	[62]
Metformin and phenformin prodrugs		Intracellular accumulation of the compounds and in vivo studies to determine MTD and tumor burden	All complexes were shown to accumulate intracellularly in a dose-dependent manner, and metformin + phenformin prodrugs decreased tumor burden with MTD determined as 15 mg/kg.	[63]
Lipophilic salts of metformin		Solubility and permeation	Metformin docusate exerted greater anticancer effects due to its enhanced lipophilicity and lipid solubility properties.	[64]
Metformin, cisplatin, and gefitinib		Cell viability, hypoxia gene expression, and CSC population were studied through luciferase activity	Greater reduction in cell viability, HIF activity, and CSC population was seen in the cisplatin + metformin + gefitinib (CMG) group as compared to individual or two-agent combination.	[65]
Metformin and 2-DG		Exploring the differential expression of genes with glucose starvation or glucose-lowering agents such as 2-DG or metformin	The glucose starvation environment was most effective in damaging cancerous cells as compared to 2-DG and metformin.	[66]
Metformin	TRAIL	Expression of TRAIL, cell viability	The apoptotic process was triggered by the increased expression of TRAIL. Higher metformin concentrations were associated with increased TRAIL protein levels, and the inhibition of TRAIL led to the attenuation of metformin-induced apoptosis.	[67]
Metformin		Metformin and its relationship to metformin transporters named organic cation transporter 3 (OCT3)	Metformin was more efficacious in OCT3-MCF7 cells as compared to MCF-7 cells.	[68]
Metformin and naringenin		Cell viability	The cotreatment with both metformin and naringenin resulted in a significant reduced cell viability.	[69]
Metformin and TRAIL receptor agonists	TRAIL, TRAIL-R2 mRNA	TRAIL receptor sensitization to agonists, caspase activation, receptor expression over cell surface, and X-linked inhibitor of apoptosis protein (XIAP) expression and in vivo studies	Metformin sensitized TNBC cell lines to TRAIL agonists via inducing caspase activation. Real-time PCR results showed only a slight increase in TRAIL-R2 mRNA levels. In vitro investigation was consistent with the in vivo testing as the combination group had reduced XIAP protein levels. Combination treatment inhibited lung metastases to a similar degree to that in TRAIL alone.	[70]
Metformin and hemin	Metabolism, extracellular acidification rate (ECAR), oxygen consumption rate (OCR)	ECAR, OCR	A notable increase in OCR and ATP levels but decreased ECAR and intermediate compounds of the glycolysis pathway were noted.	[71]
Metformin and JNK signal pathway		Tumor-infiltrating lymphocytes, cell viability	Higher concentrations of metformin-suppressed cell viability.	[72]
Metformin, Fluvastatin, and aspirin		Cell-colonizing ability and in vivo studies	The synergistic combination of fluvastatin, metformin, and aspirin resulted in 100% inhibition of cell colonization.	[73]
Metformin		The role of metformin on YAP/TAZ axis regulation and its relation to achieving BC therapeutic effects	Metformin impeded cell growth in a dose-dependent manner and induced apoptosis via significantly arresting the cells in the G1 phase.	[74]
Hyaluronic acid-engrafted metformin-loaded graphene oxide (HA-GO-Met) nanoparticles		Cell viability and cell migration	HA-GO-Met nanoparticles were effective in inducing cell death and attenuating cell migration as compared to GO-Met or metformin alone.	[75]
Metformin and relaxin		Altering the immune microenvironment of tumors by reducing cancer-associated fibroblasts	Coumarins with LPP remained on the edges of the tumor sphere while those with LPPR were efficient in penetrating deep into the tumor, which indicates the efficiency of pRLN in increasing penetration.	[76]

**Table 2:** Evidence of metformin and metformin-based approaches from clinical studies is summarized.

Agents	Parameters	Findings	Refs.
Metformin and erlotinib	MTD, DLT, response rate, stable disease rate, and PFS	Absence of sufficient clinical benefits of this combination.	[84]
Metformin	Fasting insulin, glucose, CRP, estradiol, testosterone, and SHBG	Fasting insulin, estradiol, testosterone, and SHBG concentrations were improved by metformin. In addition, while metformin resulted in a higher average weight loss, glucose and CRP were unaffected as compared to placebo.	[85]
Metformin	Statistical insights	Women using metformin with T2D were at increased risk of developing TNBC.	[86]
Metformin	Relationship between T2D, use of metformin, and BC risk	T2D patients treated with metformin were found to have an increased risk of TNBC.	[87]

**Table 3:** Summary of ongoing and completed clinical trials of metformin in TNBC.

Study Title	Study Phase (Status)	Conditions	Intervention	Primary Outcome Measures	NCT Number	Refs.
Calorie Restriction With or Without Metformin in Triple-Negative Breast Cancer	Phase II (Unknown)	TNBC	Fasting-mimicking diet, Metformin, Preoperative Chemotherapy	Rate of pathologic complete responses.	NCT04248998	[88]
The Study of Quadruple Therapy Quercetin, Zinc, Metformin, and EGCG as Adjuvant Therapy for Early, Metastatic Breast Cancer and Triple-Negative Breast Cancer, a Novel Mechanism	Phase I (Completed)	TNBC	Combination product of Quercetin, EGCG, metformin, zinc	Invasive disease-free survival at 3 years.	NCT05680662	[89]
Study of Erlotinib and Metformin in Triple-Negative Breast Cancer	Phase I (Completed)	TNBC	Erlotinib in combination with metformin	Maximum tolerated dose of metformin in combination with a fixed dose of 150 mg erlotinib daily.	NCT01650506	[90]
Alpelisib/iNOS Inhibitor/Nab-paclitaxel in Patients With HER2 Negative Metaplastic Breast Cancer (MpBC)	Phase II (Recruiting)	HER2-negative BC, MBC, Metaplastic breast carcinoma, TNBC	L-NMMA	Define recommended phase II dose and objective response rate.	NCT05660083	[91]
I-SPY TRIAL: Neoadjuvant and Personalized Adaptive Novel Agents to Treat Breast Cancer	Phase II (Recruiting)	Breast neoplasms, BC, Breast tumors, TNBC, HER2-positive BC, HER2-negative BC, Hormone receptor positive tumor, Hormone receptor negative tumor, early-stage BC, Locally advanced BC	Standard therapy, AMG 386 with or without Trastuzumab, AMG 479 (Ganitumab) plus metformin, and others	Determine whether adding experimental agents to standard neoadjuvant medications increases the probability of pathologic complete response over standard neoadjuvant chemotherapy for each biomarker signature established at trial entry.	NCT01042379	[92]

## Data Availability

The summary of the data is included in this manuscript.

## Abbreviations

BC	Breast cancer
TNBC	Triple-negative breast cancer
ER	Estrogen
PR	Progesterone
HER2	Human epidermal growth factor receptor 2
T2D	Type 2 diabetes
IGF-1	Insulin-like growth factor 1
TNF-α	Tumor necrosis factor-α
IRS1	Insulin receptor substrate
ROS	Reactive oxygen species
IR	Insulin receptor
IGFBP-1	IGF-Binding Protein 1
2-DG	2-deoxyglucose
GLUT	Glucose transport proteins
TNB	Triple-Negative B
TNA	Triple-Negative A
EMT	Epithelial–mesenchymal transition
PI3KI	Phosphatidylinositol-3-kinase inhibitor
IGF-1R	Insulin-like growth factor 1 receptor
CI	Combination index
RPPA	Reverse phase protein lysate microarray
MTD	Maximum tolerated dose
CSC	Cancer stem cells
CMG	Metformin and gefitinib
ALDH+	Aldehyde dehydrogenase
HIF	Hypoxia-inducible factor
TRAIL	TNF-related apoptosis-inducing ligand
XIAP	X-linked inhibitor of apoptosis protein
RLN	Relaxin
BACH1	BTB and CNC homology 1
ECAR	Metabolism, extracellular acidification rate
OCR	Oxygen consumption rate
OCT3	Organic cation transporter 3
CRP	C-reactive protein
SHBG	Sex hormone-binding globulin

## References

[1] Giaquinto A.N., Sung H., Miller K.D., Kramer J.L., Newman L.A., Minihan A., Jemal A., Siegel R.L. (2022). Breast Cancer Statistics, 2022. CA Cancer J. Clin..

[2] Siegel R.L., Miller K.D., Wagle N.S., Jemal A. (2023). Cancer Statistics, 2023. CA Cancer J. Clin..

[3] Siegel R.L., Miller K.D., Jemal A. (2017). Cancer Statistics, 2017. CA Cancer J. Clin..

[4] Bray F., Ferlay J., Soerjomataram I., Siegel R.L., Torre L.A., Jemal A. (2018). Global Cancer Statistics 2018: GLOBOCAN Estimates of Incidence and Mortality Worldwide for 36 Cancers in 185 Countries. CA Cancer J. Clin..

[5] Yeo S.K., Guan J.L. (2017). Breast Cancer: Multiple Subtypes within a Tumor?. Trends Cancer.

[6] Feng Y., Spezia M., Huang S., Yuan C., Zeng Z., Zhang L., Ji X., Liu W., Huang B., Luo W. (2018). Breast Cancer Development and Progression: Risk Factors, Cancer Stem Cells, Signaling Pathways, Genomics, and Molecular Pathogenesis. Genes Dis..

[7] Sirhan Z., Thyagarajan A., Sahu R.P. (2022). The Efficacy of Tucatinib-Based Therapeutic Approaches for HER2-Positive Breast Cancer. Mil. Med. Res..

[8] Wang Q., Xu M., Sun Y., Chen J., Chen C., Qian C., Chen Y., Cao L., Xu Q., Du X. (2019). Gene Expression Profiling for Diagnosis of Triple-Negative Breast Cancer: A Multicenter, Retrospective Cohort Study. Front. Oncol..

[9] Kumar P., Aggarwal R. (2016). An Overview of Triple-Negative Breast Cancer. Arch. Gynecol. Obstet..

[10] Derakhshan F., Reis-Filho J.S. (2022). Pathogenesis of Triple-Negative Breast Cancer. Annu. Rev. Pathol. Mech. Dis..

[11] Baranova A., Krasnoselskyi M., Starikov V., Kartashov S., Zhulkevych I., Vlasenko V., Oleshko K., Bilodid O., Sadchikova M., Vinnyk Y. (2022). Triple-Negative Breast Cancer: Current Treatment Strategies and Factors of Negative Prognosis. J. Med. Life.

[12] Maqbool M., Bekele F., Fekadu G. (2022). Treatment Strategies Against Triple-Negative Breast Cancer: An Updated Review. Breast Cancer Targets Ther..

[13] Lin X., Xu Y., Pan X., Xu J., Ding Y., Sun X., Song X., Ren Y., Shan P.F. (2020). Global, Regional, and National Burden and Trend of Diabetes in 195 Countries and Territories: An Analysis from 1990 to 2025. Sci. Rep..

[14] Giovannucci E., Harlan D.M., Archer M.C., Bergenstal R.M., Gapstur S.M., Habel L.A., Pollak M., Regensteiner J.G., Yee D. (2010). Diabetes and Cancer: A Consensus Report. Diabetes Care.

[15] Samuel S.M., Varghese E., Varghese S., Büsselberg D. (2018). Challenges and Perspectives in the Treatment of Diabetes Associated Breast Cancer. Cancer Treat. Rev..

[16] Min W., Wang B., Guo A., Mao G., Zhao Y., Zhang S., He R., Min Y., Huang Y. (2020). The Effect of Metformin on the Clinicopathological Features of Breast Cancer with Type 2 Diabetes. World J. Oncol..

[17] Cejuela M., Martin-Castillo B., Menendez J.A., Pernas S. (2022). Metformin and Breast Cancer: Where Are We Now?. Int. J. Mol. Sci..

[18] Corleto K.A., Strandmo J.L., Giles E.D. (2024). Metformin and Breast Cancer: Current Findings and Future Perspectives from Preclinical and Clinical Studies. Pharmaceuticals.

[19] Lord S.R., Harris A.L. (2023). Is It Still Worth Pursuing the Repurposing of Metformin as a Cancer Therapeutic?. Br. J. Cancer.

[20] Wahdan-Alaswad R.S., Edgerton S.M., Salem H.S., Thor A.D. (2018). Metformin Targets Glucose Metabolism in Triple Negative Breast Cancer. J. Oncol. Transl. Res..

[21] Jordt N., Kjærgaard K.A., Thomsen R.W., Borgquist S., Cronin-Fenton D. (2023). Breast Cancer and Incidence of Type 2 Diabetes Mellitus: A Systematic Review and Meta-Analysis. Breast Cancer Res. Treat..

[22] Matou-Nasri S., Aldawood M., Alanazi F., Khan A.L. (2023). Updates on Triple-Negative Breast Cancer in Type 2 Diabetes Mellitus Patients: From Risk Factors to Diagnosis, Biomarkers and Therapy. Diagnostics.

[23] Zhang F., de Haan-Du J., Sidorenkov G., Landman G.W.D., Jalving M., Zhang Q., de Bock G.H. (2021). Type 2 Diabetes Mellitus and Clinicopathological Tumor Characteristics in Women Diagnosed with Breast Cancer: A Systematic Review and Meta-Analysis. Cancers.

[24] Eketunde A.O. (2020). Diabetes as a Risk Factor for Breast Cancer. Cureus.

[25] Bashraheel S.S., Kheraldine H., Khalaf S., Al Moustafa A.E. (2023). Metformin and HER2-Positive Breast Cancer: Mechanisms and Therapeutic Implications. Biomed. Pharmacother..

[26] Christopoulos P.F., Msaouel P., Koutsilieris M. (2015). The Role of the Insulin-like Growth Factor-1 System in Breast Cancer. Mol. Cancer.

[27] Durrani I.A., Bhatti A., John P. (2021). The Prognostic Outcome of ‘Type 2 Diabetes Mellitus and Breast Cancer’ Association Pivots on Hypoxia-Hyperglycemia Axis. Cancer Cell Int..

[28] Kurelac I., Umesh Ganesh N., Iorio M., Porcelli A.M., Gasparre G. (2020). The Multifaceted Effects of Metformin on Tumor Microenvironment. Semin. Cell Dev. Biol..

[29] Samuel S.M., Varghese E., Koklesová L., Líšková A., Kubatka P., Büsselberg D. (2020). Counteracting Chemoresistance with Metformin in Breast Cancers: Targeting Cancer Stem Cells. Cancers.

[30] Yee L.D., Mortimer J.E., Natarajan R., Dietze E.C., Seewaldt V.L. (2020). Metabolic Health, Insulin, and Breast Cancer: Why Oncologists Should Care About Insulin. Front. Endocrinol..

[31] Khan I., Kamal A., Akhtar S. (2024). Diabetes Driven Oncogenesis and Anticancer Potential of Repurposed Antidiabetic Drug: A Systemic Review. Cell Biochem. Biophys..

[32] García-Estévez L., Cortés J., Pérez S., Calvo I., Gallegos I., Moreno-Bueno G. (2021). Obesity and Breast Cancer: A Paradoxical and Controversial Relationship Influenced by Menopausal Status. Front. Oncol..

[33] Sahu P., Camarillo I.G., Sundararajan R. (2024). Efficacy of Metformin and Electrical Pulses in Breast Cancer MDA-MB-231 Cells. Explor. Target. Antitumor Ther..

[34] Mallik R., Chowdhury T.A. (2018). Metformin in Cancer. Diabetes Res. Clin. Pract..

[35] Ng M., Fleming T., Robinson M., Thomson B., Graetz N., Margono C., Mullany E.C., Biryukov S., Abbafati C., Abera S.F. (2014). Global, Regional, and National Prevalence of Overweight and Obesity in Children and Adults during 1980-2013: A Systematic Analysis for the Global Burden of Disease Study 2013. Lancet.

[36] Goodwin P.J., Stambolic V. (2015). Impact of the Obesity Epidemic on Cancer. Annu. Rev. Med..

[37] Song J., Du J., Han L., Lin X., Fan C., Chen G. (2023). The Effect of Metformin on Triple-Negative Breast Cancer Cells and Nude Mice ORIGINAL RESEARCH. Altern. Ther..

[38] Lyu X., Zhang Q., Fares H.M., Wang Y., Han Y., Sun L. (2022). Contribution of Adipocytes in the Tumor Microenvironment to Breast Cancer Metabolism. Cancer Lett..

[39] Verras G.I., Tchabashvili L., Chlorogiannis D.D., Mulita F., Argentou M.I. (2023). Updated Clinical Evidence on the Role of Adipokines and Breast Cancer: A Review. Cancers.

[40] Georgiou G.P., Provatopoulou X., Kalogera E., Siasos G., Menenakos E., Zografos G.C., Gounaris A. (2016). Serum Resistin Is Inversely Related to Breast Cancer Risk in Premenopausal Women. Breast.

[41] Niu J., Jiang L., Guo W., Shao L., Liu Y., Wang L. (2013). The Association between Leptin Level and Breast Cancer: A Meta-Analysis. PLoS ONE.

[42] Khan S., Shukla S., Sinha S., Meeran S.M. (2013). Role of Adipokines and Cytokines in Obesity-Associated Breast Cancer: Therapeutic Targets. Cytokine Growth Factor Rev..

[43] Koprivčić I., Marjanović K., Matić A., Levak M.T., Lovrić I., Pauzar B., Erić I., Wertheimer V. (2022). SERUM LEPTIN LEVEL IN BREAST CANCER. Acta Clin. Croat..

[44] Samson S.L., Vellanki P., Blonde L., Christofides E.A., Galindo R.J., Hirsch I.B., Isaacs S.D., Izuora K.E., Low Wang C.C., Twining C.L. (2023). American Association of Clinical Endocrinology Consensus Statement: Comprehensive Type 2 Diabetes Management Algorithm—2023 Update. Endocr. Pract..

[45] Nedeljković M., Damjanović A. (2019). Mechanisms of Chemotherapy Resistance in Triple-Negative Breast Cancer-How We Can Rise to the Challenge. Cells.

[46] Mahmoudi G., Ehteshaminia Y., Kokhaei P., Jalali S.F., Jadidi-Niaragh F., Pagheh A.S., Enderami S.E., Kenari S.A., Hassannia H. (2024). Enhancement of Targeted Therapy in Combination with Metformin on Human Breast Cancer Cell Lines. Cell Commun. Signal..

[47] Foretz M., Guigas B., Viollet B. (2023). Metformin: Update on Mechanisms of Action and Repurposing Potential. Nat. Rev. Endocrinol..

[48] Alimova I.N., Liu B., Fan Z., Edgerton S.M., Dillon T., Lind S.E., Thor A.D. (2009). Metformin Inhibits Breast Cancer Cell Growth, Colony Formation and Induces Cell Cycle Arrest in Vitro. Cell Cycle.

[49] Stoian A.M.P., Rizzo M. (2020). Metformin.

[50] Deng X.S., Wang S., Deng A., Liu B., Edgerton S.M., Lind S.E., Wahdan-Alaswad R., Thor A.D. (2012). Metformin Targets Stat3 to Inhibit Cell Growth and Induce Apoptosis in Triple-Negative Breast Cancers. Cell Cycle.

[51] Amaral I., Silva C., Correia-Branco A., Martel F. (2018). Effect of Metformin on Estrogen and Progesterone Receptor-Positive (MCF-7) and Triple-Negative (MDA-MB-231) Breast Cancer Cells. Biomed. Pharmacother..

[52] Cingir Koker S., Yalcin B., Dogan Turacli I. (2022). Metformin Resistant MDA-MB-468 Cells Exhibit EMT-like Phenotype and Increased Migration Capacity. Mol. Biol. Rep..

[53] Li Y., Zhang Q., Yang J., He W., Jiang Y., Chen Y., Wang Y. (2023). Metformin Combined with Glucose Starvation Synergistically Suppress Triple-Negative Breast Cancer by Enhanced Unfolded Protein Response. Biochem. Biophys. Res. Commun..

[54] Samuel S.M., Varghese E., Satheesh N.J., Triggle C.R., Büsselberg D. (2023). Metabolic Heterogeneity in TNBCs: A Potential Determinant of Therapeutic Efficacy of 2-Deoxyglucose and Metformin Combinatory Therapy. Biomed. Pharmacother..

[55] Rico M., Baglioni M., Bondarenko M., Laluce N.C., Rozados V., André N., Carré M., Scharovsky O.G., Márquez M.M. (2017). Metformin and Propranolol Combination Prevents Cancer Progression and Metastasis in Different Breast Cancer Models. Oncotarget.

[56] Anselmino L.E., Baglioni M.V., Malizia F., Laluce N.C., Etichetti C.B., Marignac V.L.M., Rozados V., Scharovsky O.G., Girardini J., Rico M.J. (2021). Repositioning Metformin and Propranolol for Colorectal and Triple Negative Breast Cancers Treatment. Sci. Rep..

[57] Repas J., Zupin M., Vodlan M., Veranič P., Gole B., Potočnik U., Pavlin M. (2022). Dual Effect of Combined Metformin and 2-Deoxy-D-Glucose Treatment on Mitochondrial Biogenesis and PD-L1 Expression in Triple-Negative Breast Cancer Cells. Cancers.

[58] Tan X., Li Y., Hou Z., Zhang M., Li L., Wei J. (2023). Combination Therapy with PD-1 Inhibition plus Rapamycin and Metformin Enhances Anti-Tumor Efficacy in Triple Negative Breast Cancer. Exp. Cell Res..

[59] Xue L., Chen F., Yue F., Camacho L., Kothapalli S., Wei G., Huang S., Mo Q., Ma F., Li Y. (2021). Metformin and an Insulin/IGF-1 Receptor Inhibitor Are Synergistic in Blocking Growth of Triple-Negative Breast Cancer. Breast Cancer Res. Treat..

[60] Cheng T., Wang C., Lu Q., Cao Y., Yu W., Li W., Liu B., Gao X., Lü J., Pan X. (2022). Metformin Inhibits the Tumor-Promoting Effect of Low-Dose Resveratrol, and Enhances the Anti-Tumor Activity of High-Dose Resveratrol by Increasing Its Reducibility in Triple Negative Breast Cancer. Free Radic. Biol. Med..

[61] Lee J.O., Kang M.J., Byun W.S., Kim S.A., Seo I.H., Han J.A., Moon J.W., Kim J.H., Kim S.J., Lee E.J. (2019). Metformin Overcomes Resistance to Cisplatin in Triple-Negative Breast Cancer (TNBC) Cells by Targeting RAD51. Breast Cancer Res..

[62] Sahu P., Camarillo I.G., Sundararajan R. (2022). Enhanced Antiproliferation Potency of Electrical Pulse-Mediated Metformin and Cisplatin Combination Therapy on MDA-MB-231 Cells. Appl. Biochem. Biotechnol..

[63] Babak M.V., Chong K.R., Rapta P., Zannikou M., Tang H.M., Reichert L., Chang M.R., Kushnarev V., Heffeter P., Meier-Menches S.M. (2021). Interfering with Metabolic Profile of Triple-Negative Breast Cancers Using Rationally Designed Metformin Prodrugs. Angew. Chem.—Int. Ed..

[64] Saeed H.K., Sutar Y., Patel P., Bhat R., Mallick S., Hatada A.E., Koomoa D.L.T., Lange I., Date A.A. (2021). Synthesis and Characterization of Lipophilic Salts of Metformin to Improve Its Repurposing for Cancer Therapy. ACS Omega.

[65] Sulaiman A., McGarry S., Chambers J., Al-Kadi E., Phan A., Li L., Mediratta K., Dimitroulakos J., Addison C., Li X. (2020). Targeting Hypoxia Sensitizes TNBC to Cisplatin and Promotes Inhibition of Both Bulk and Cancer Stem Cells. Int. J. Mol. Sci..

[66] Aoun R., El Hadi C., Tahtouh R., El Habre R., Hilal G. (2022). Microarray Analysis of Breast Cancer Gene Expression Profiling in Response to 2-Deoxyglucose, Metformin, and Glucose Starvation. Cancer Cell Int..

[67] Liu S., Polsdofer E.V., Zhou L., Ruan S., Lyu H., Hou D., Liu H., Thor A.D., He Z., Liu B. (2021). Upregulation of Endogenous TRAIL-Elicited Apoptosis Is Essential for Metformin-Mediated Antitumor Activity against TNBC and NSCLC. Mol. Ther. Oncolytics.

[68] Cai H., Everett R.S., Thakker D.R. (2019). Efficacious Dose of Metformin for Breast Cancer Therapy Is Determined by Cation Transporter Expression in Tumours. Br. J. Pharmacol..

[69] Pateliya B., Burade V., Goswami S. (2021). Combining Naringenin and Metformin with Doxorubicin Enhances Anticancer Activity against Triple-Negative Breast Cancer in Vitro and in Vivo. Eur. J. Pharmacol..

[70] Strekalova E., Malin D., Rajanala H., Cryns V.L. (2017). Metformin Sensitizes Triple-Negative Breast Cancer to Proapoptotic TRAIL Receptor Agonists by Suppressing XIAP Expression. Breast Cancer Res. Treat..

[71] Lee J., Yesilkanal A.E., Wynne J.P., Frankenberger C., Liu J., Yan J., Elbaz M., Rabe D.C., Rustandy F.D., Tiwari P. (2019). Effective Breast Cancer Combination Therapy Targeting BACH1 and Mitochondrial Metabolism. Nature.

[72] Wang R., Li Y., Zhao Y., Shi F., Zhou Q., Wu J., Lyu S., Song Q. (2022). Metformin Inducing the Change of Functional and Exhausted Phenotypic Tumor-Infiltrated Lymphocytes and the Correlation with JNK Signal Pathway in Triple-Negative Breast Cancer. Breast Cancer Targets Ther..

[73] Bhardwaj A., Singh H., Trinidad C.M., Albarracin C.T., Hunt K.K., Bedrosian I. (2018). The IsomiR-140-3p-Regulated Mevalonic Acid Pathway as a Potential Target for Prevention of Triple Negative Breast Cancer. Breast Cancer Res..

[74] Xu Y., Cai H., Xiong Y., Tang L., Li L., Zhang L., Shen Y., Yang Y., Lin L., Huang J. (2023). YAP/TAZ Axis Was Involved in the Effects of Metformin on Breast Cancer. J. Chemother..

[75] Basu A., Upadhyay P., Ghosh A., Bose A., Gupta P., Chattopadhyay S., Chattopadhyay D., Adhikary A. (2021). Hyaluronic Acid Engrafted Metformin Loaded Graphene Oxide Nanoparticle as CD44 Targeted Anti-Cancer Therapy for Triple Negative Breast Cancer. Biochim. Biophys. Acta Gen. Subj..

[76] Zhang H., Chen L., Zhao Y., Luo N., Shi J., Xu S., Ma L., Wang M., Gu M., Mu C. (2023). Relaxin-Encapsulated Polymeric Metformin Nanoparticles Remodel Tumor Immune Microenvironment by Reducing CAFs for Efficient Triple-Negative Breast Cancer Immunotherapy. Asian J. Pharm. Sci..

[77] Park J.H., Vithayathil S., Kumar S., Sung P.L., Dobrolecki L.E., Putluri V., Bhat V.B., Bhowmik S.K., Gupta V., Arora K. (2016). Fatty Acid Oxidation-Driven Src Links Mitochondrial Energy Reprogramming and Oncogenic Properties in Triple-Negative Breast Cancer. Cell Rep..

[78] Attri K.S., Park J.H., Kaipparettu B.A. (2022). Redox Regulation of Hybrid Metabolic State in Breast Cancer Metastasis. Ann. Transl. Med..

[79] Jia D., Park J.H., Jung K.H., Levine H., Kaipparettu B.A. (2018). Elucidating the Metabolic Plasticity of Cancer: Mitochondrial Reprogramming and Hybrid Metabolic States. Cells.

[80] Tan A.S., Baty J.W., Dong L.F., Bezawork-Geleta A., Endaya B., Goodwin J., Bajzikova M., Kovarova J., Peterka M., Yan B. (2015). Mitochondrial Genome Acquisition Restores Respiratory Function and Tumorigenic Potential of Cancer Cells without Mitochondrial DNA. Cell Metab..

[81] Weinberg S.E., Chandel N.S. (2015). Targeting Mitochondria Metabolism for Cancer Therapy. Nat. Chem. Biol..

[82] Xu Q., Biener-Ramanujan E., Yang W., Ramanujan V.K. (2015). Targeting Metabolic Plasticity in Breast Cancer Cells via Mitochondrial Complex I Modulation. Breast Cancer Res. Treat..

[83] Park J.H., Jung K.H., Jia D., Yang S., Attri K.S., Ahn S., Murthy D., Samanta T., Dutta D., Ghidey M. (2025). Biguanides Antithetically Regulate Tumor Properties by the Dose-Dependent Mitochondrial Reprogramming-Driven c-Src Pathway. Cell Rep. Med..

[84] Fenn K., Maurer M., Lee S.M., Crew K.D., Trivedi M.S., Accordino M.K., Hershman D.L., Kalinsky K. (2020). Phase 1 Study of Erlotinib and Metformin in Metastatic Triple-Negative Breast Cancer. Clin. Breast Cancer.

[85] Patterson R.E., Marinac C.R., Sears D.D., Kerr J., Hartman S.J., Cadmus-Bertram L., Villaseñor A., Flatt S.W., Godbole S., Li H. (2018). The Effects of Metformin and Weight Loss on Biomarkers Associated with Breast Cancer Outcomes. J. Natl. Cancer Inst..

[86] Chen H., Cook L.S., Tang M.T.C., Hill D.A., Wiggins C.L., Li C.I. (2019). Relationship between Diabetes and Diabetes Medications and Risk of Different Molecular Subtypes of Breast Cancer. Cancer Epidemiol. Biomark. Prev..

[87] Park Y.M.M., Bookwalter D.B., O’Brien K.M., Jackson C.L., Weinberg C.R., Sandler D.P. (2021). A Prospective Study of Type 2 Diabetes, Metformin Use, and Risk of Breast Cancer. Ann. Oncol..

[88] Fondazione IRCCS Istituto Nazionale dei Tumori, Milano (2023). Calorie Restriction With or Without Metformin in Triple Negative Breast Cancer (BREAKFAST). ClinicalTrials.gov.

[89] Ministry of Health, Saudi Arabia (2023). The Study of Quadruple Therapy Quercetin, Zinc, Metformin, and EGCG as Adjuvant Therapy for Early, Metastatic Breast Cancer and Triple-negative Breast Cancer, a Novel Mechanism. ClinicalTrials.gov.

[90] Columbia University (2017). Study of Erlotinib and Metformin in Triple Negative Breast Cancer. ClinicalTrials.gov.

[91] The Methodist Hospital Research Institute (2025). Alpelisib/iNOS Inhibitor/Nab-paclitaxel in Patients with HER2 Negative Metaplastic Breast Cancer (MpBC). ClinicalTrials.gov.

[92] QuantumLeap Healthcare Collaborative (2024). I-SPY TRIAL: Neoadjuvant and Personalized Adaptive Novel Agents to Treat Breast Cancer (I-SPY). ClinicalTrials.gov.

